# Transcriptomic changes in an animal-bacterial symbiosis under modeled microgravity conditions

**DOI:** 10.1038/srep46318

**Published:** 2017-04-10

**Authors:** Giorgio Casaburi, Irina Goncharenko-Foster, Alexandrea A. Duscher, Jamie S. Foster

**Affiliations:** 1 Department of Microbiology and Cell Science, University of Florida, Space Life Science Lab, Merritt Island, FL, USA

## Abstract

Spaceflight imposes numerous adaptive challenges for terrestrial life. The reduction in gravity, or microgravity, represents a novel environment that can disrupt homeostasis of many physiological processes. Additionally, it is becoming increasingly clear that an organism’s microbiome is critical for host health and examining its resiliency in microgravity represents a new frontier for space biology research. In this study, we examine the impact of microgravity on the interactions between the squid *Euprymna scolopes* and its beneficial symbiont *Vibrio fischeri*, which form a highly specific binary mutualism. First, animals inoculated with *V. fischeri* aboard the space shuttle showed effective colonization of the host light organ, the site of the symbiosis, during space flight. Second, RNA-Seq analysis of squid exposed to modeled microgravity conditions exhibited extensive differential gene expression in the presence and absence of the symbiotic partner. Transcriptomic analyses revealed in the absence of the symbiont during modeled microgravity there was an enrichment of genes and pathways associated with the innate immune and oxidative stress response. The results suggest that *V. fischeri* may help modulate the host stress responses under modeled microgravity. This study provides a window into the adaptive responses that the host animal and its symbiont use during modeled microgravity.

Spaceflight impacts all living organisms from their genome to physiome. The physical factors associated with the space environment present a unique set of stresses on biological systems. Of these physical factors the reduction in gravity, or microgravity, is one of the most widely studied^1^,^2^,^3^,^4^. The large-scale physiological effects of microgravity on animals are relatively well known. For example in humans, microgravity conditions result in bone loss, upwards to 3% per month^5^, permutations to both the adaptive and innate immune systems^3^,^6^,^7^, and a potential increased risk of bacterial and viral infections^8^,^9^,^10^,^11^,^12^,^13^. Although the overarching phenotypes associated with microgravity exposure have been observed for decades, the underlying etiology of these effects at the cellular and biomolecular level are not yet fully delineated.

Compounding our understanding of the mechanisms underlying these physiological effects is the relatively unknown impact of microgravity on the microbiome associated with eukaryotic organisms. Over the past decade there has been a paradigm shift in our understanding of the importance the microbiome plays in maintaining host health^14^,^15^. Aspects previously attributed to the host are now recognized as being the result of interactions with microbes. For example, analyses of germ-free and normal mice have shown that the gut microbiota is responsible for most of the metabolites found in mammalian blood^16^. Additionally, some species of bacteria within the human gut have been recently correlated to the onset of several autoimmune diseases, such as rheumatoid arthritis and Type 1 diabetes^17^,^18^, as well as inflammatory bowel disease^19^,^20^ and colorectal cancers^21^ suggesting that perturbations to the gut environment can increase the risk of disease. Although the short- and long-term resilience of animal microbiomes in response to microgravity is virtually unknown, microbial exchange and transfers do occur between host and its microbiome during spaceflight^22^,^23^,^24^. Assessing the impact of microgravity on a host organism and its microbiota requires a comprehensive analysis that includes the study of both microbes and their hosts *in situ* to fully understand the requirements needed to maintain host health in the space environment.

To examine the impact of microgravity on beneficial host-microbe interactions *in situ*, the symbiosis between the Hawaiian bobtail squid *Euprymna scolopes* and its bioluminescent partner *Vibrio fischeri* has emerged as tractable model for space biology research^24^,^25^,^26^,^27^. For almost 30 years, this mutualism has enabled the elucidation of fundamental principles associated with horizontally acquired symbioses and their influence on normal animal development^28^,^29^,^30^. The symbionts colonize a specialized light organ located within the mantle cavity (Fig. 1a–d). On either side of the light organ there is a single layer of ciliated epithelial cells that form appendage-like structures that draw in bacteria-enriched water from the environment (Fig. 1b). Upon hatching, the superficial ciliated epithelial appendages (CEA) secrete mucus that facilitates the attachment of 3–5 *V. fischeri* cells^31^,^32^,^33^. After binding to the cilia, *V. fischeri* begin to aggregate near the CEA in which they outcompete other microbes and begin to migrate to pores on the surface of the CEA (Fig. 1d,e), travel down ciliated ducts, and enter one of three epithelial-lined crypt spaces (Fig. 1e)^34^,^35^.

Once the colonization process has been initiated a series of *V. fischeri*-induced developmental changes occur in the light organ that include the apoptotic cell death (Fig. 1d), regression of the CEA structures and remodeling of the epithelial cells that line the crypt spaces^36^,^37^,^38^,^39^. In addition to these morphogenic changes the binding and aggregation of *V. fischeri* outside the light organ activates the host immune response, principally the movement of hemocytes (i.e., invertebrate macrophage-like cells), into the blood sinus of the CEA as early as 2 h post-inoculation^40^. Hemocytes also infiltrate the crypt spaces after colonization and by 36 h there is a significant increase in the number of these macrophage-like cells compared to aposymbiotic animals and by 48 h they exhibit phagocytic uptake of bacteria^40^,^41^.

Under modeled microgravity conditions, however, many of these bacteria-induced developmental events are altered^26^,^27^. For example, the trafficking of the hemocytes into the CEA blood sinus is delayed by 10 h in symbiotic animals, and the number of hemocytes within the sinus never reaches levels observed in gravity controls^26^. Additionally, the onset of apoptosis and regression of the CEA structures after colonization is accelerated compared to the normal developmental timeline under simulated microgravity conditions^26^. Although there are clear phenotypes associated with the modeled microgravity, many of the underlying molecular changes are unknown. In this study, we begin to characterize the genetic architecture of the host response to low-shear modeled microgravity by sequencing the transcriptome of the light organ in the presence and absence of their mutualistic symbionts. Both the host and bacteria were co-incubated within high-aspect-ratio rotating vessels (HARVs) that effectively mimic the low shear, low turbulent nature of the space environment^42^,^43^. Additionally, we examined light organ morphology of animals exposed to natural space conditions aboard the space shuttle to demonstrate that *V. fischeri* can effectively colonize the host light organ rendering it a useful model for microgravity-related studies. Together, the results help elucidate the critical role that beneficial microbes may play in maintaining homeostasis and/or health of their host organisms during space flight.

## Results

### *Vibrio fischeri* colonize host light organ during spaceflight

**Figure 1:**
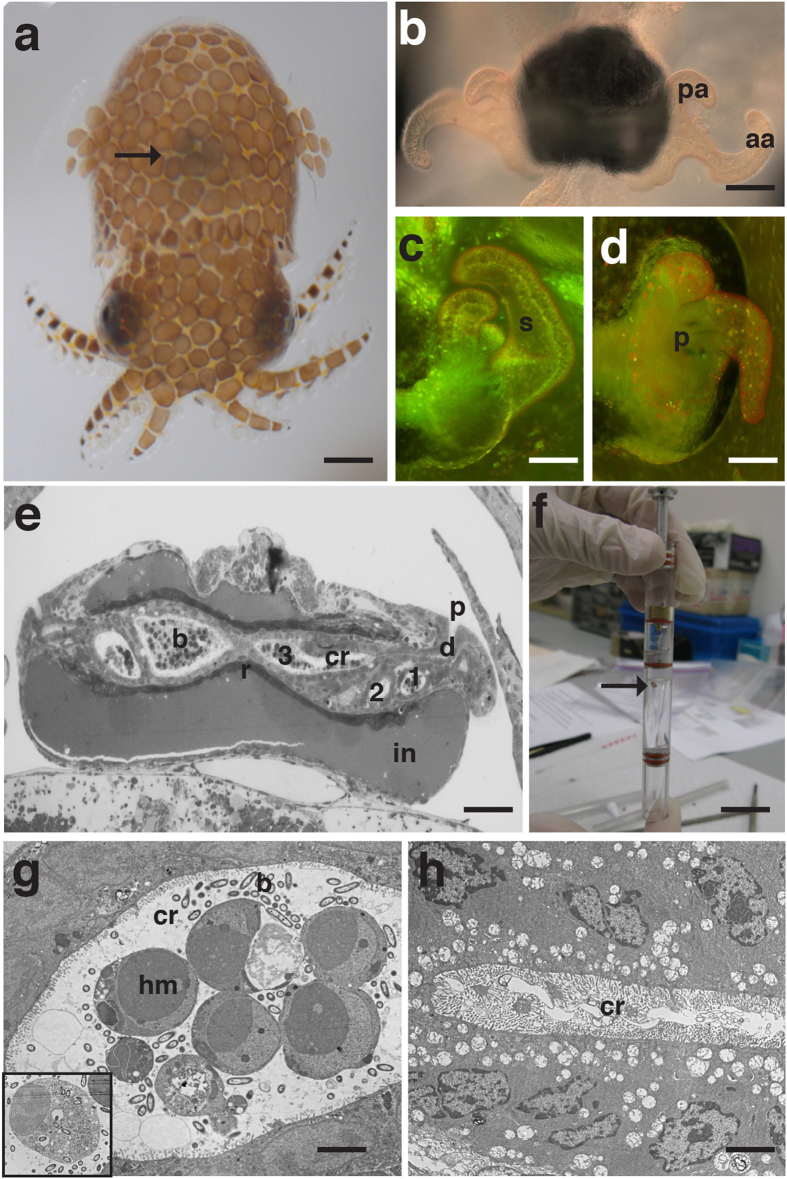
Overview of the morphology of the host light organ. (**a**) Juvenile *E. scolopes*. Arrow indicates the location of light organ. Bar = 300 μm. (**b**) Light micrograph of hatchling light organ depicting anterior (aa) and posterior appendage (pa) of the ciliated epithelial fields found on the surface of the light organ. Bar = 50 μm. (**c**) Acridine orange stained micrograph of one half of light organ in 24 h aposymbiotic animal depicting the blood sinus (s) within the ciliated epithelial appendages. Bar = 50 μm. (**d**) Acridine orange stained microgravity of half of a 24 h symbiotic light organ showing bacteria-induced apoptotic cell death and the early stages of regression of the ciliated fields and pores (p) through which the *V. fischeri* enters the light organ. (**e**) Transmission electron micrograph (TEM) of cross section of the light organ showing the entry pores (p), ciliated ducts (d) and three crypts spaces (cr 1, 2, 3) in which the *V. fischeri* (b) reside. The crypt spaces are surrounded by accessary tissues, such as the reflector (r) and ink sac (in). Bar = 25 μm (**f**) Liquid Mixing Apparatus used to house the symbiotic partners during space flight. Arrow points to juvenile squid. Bar = 1 cm. (**g**) TEM of crypt (cr) space of 23.5 h symbiotic *E. scolopes* exposed to space flight. *V. fischeri* (b) and hemocyte-like cells (hm) are present within crypt space and some show phagocytic activity of *V. fischeri* (inset represents a different micrograph). Bar = 10 μm. (**h**) TEM of 23.5 h aposymbiotic control animals exposed to space flight showing no signs of colonization in the crypt (cr) space. Bar = 10 μm.

Juvenile squid were exposed to microgravity using the Liquid Mixing Apparatus flight hardware (LMA; Fig. 1f) aboard the shuttle (STS-134 and STS-135). Transmission electron microscopy results indicated that *V. fischeri* was successfully able to colonize the host crypt spaces during space flight (Fig. 1g). Additionally, the host innate immune response was activated during the 23.5 h experimental time line during space flight. Hemocyte-like cells were observed to have infiltrated the crypt spaces (Fig. 1g) and appear to phagocytize *V. fischeri* cells (Fig. 1g inset), which is a normal component of the nascent symbiosis. The presence of the hemocytes in the flight experiments corresponded to the symbiotic ground controls (data not shown). Aposymbiotic flight animals were not colonized and showed no hemocyte trafficking into the crypt epithelial spaces (Fig. 1h).

To ensure that colonization of the host light organ under spaceflight conditions was not the product of a high dosage of *V. fischeri* (1 × 10^5^ cells per ml FSW in LMA), replicate experiments were conducted using modeled microgravity in a rotary culture system and High Aspect Ratio Vessels (HARVs) over a range of bacterial concentrations. Results indicated that even at concentrations as low as 1 × 10^3^ cells per ml, which mirrors what the host squid experience under natural conditions, *V. fischeri* was able to colonize the light organ under modeled microgravity conditions to levels observed under normal gravity controls. Plating of the host light organs on growth media showed no statistical difference in colony forming units recovered from either the modeled microgravity or gravity treatments (data not shown). Together, the flight experiment suggests that *V. fischeri* is able to effectively colonize host tissues under spaceflight conditions.

### Overview of the transcriptome of symbiotic and aposymbiotic *E. scolopes* under modeled microgravity conditions

RNA-Seq was used to evaluate changes in the host transcriptome in the presence and absence of symbiosis-competent *V. fischeri* under modeled microgravity conditions. Two developmental time points were chosen as they represent distinctive stages of light organ colonization and development. At 12 h the colonization of the light organ is newly established and luminescence is induced^44^. Additionally, 12 h is the point in which the bacteria-induced development of the light organ becomes irreversible^28^,^36^. By 24 h, key morphological changes have been initiated, such as the onset of CEA regression, increases in microvilli density and swelling of the epithelial cells lining the host crypt, as well as the initiation of mucus secretions in the crypt spaces^35^,^38^,^45^. Additionally, by 24 h more then 95% of the *V. fischeri* have become non-motile within the crypt spaces^46^. For comparison purposes, RNA-Seq libraries of nascent host squid, collected within 30 min of hatching, were also examined.

Paired-end RNA-Seq libraries using the Illumina HiSeq platform were generated in triplicate for each treatment and pooled (Table 1). After quality filtering (see Methods), an average of 50 million high quality sequences per treatment was obtained (Table 1). The reads distribution was uniform amongst the libraries generating 882,098 transcripts with a GC content of 40.77%. The raw assembly contained a median contig length of 270 bp (mean = 364 bp) with a N50 equal to 324 bp, which is comparable to other cephalopod studies^47^. Interestingly, 39,635 transcripts (or 4.5% of the total) presented more than one isoform with 487 transcripts exhibiting more than 10 different isoforms. On average, 60% of the raw reads mapped back to the generated assembly (Table 1). Although the overall extent of mapping is lower than in other model systems (e.g. mouse), the depth of sequencing and normalized average read depth (14–24 FPKM; Table 1) provides a strong foundation for comparison of differential gene expression.

### Differential expression analysis of host transcriptome under modeled microgravity conditions

**Figure 2:**
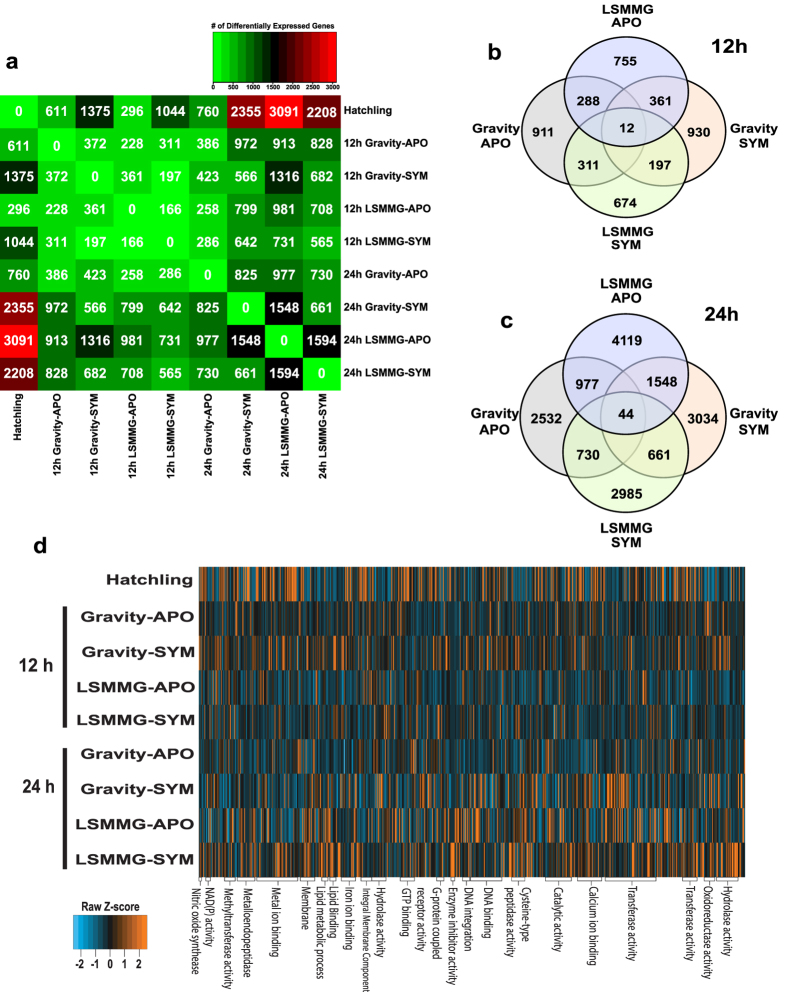
Transcriptomic analysis of hatchling, aposymbiotic (APO) and symbiotic (SYM) squid exposed to low shear modeled microgravity (LSMMG) and gravity conditions. (**a**) Matrix of the significant differentially expressed genes (DEGs) between the nine treatments. Venn diagrams comparing shared DEGs between the treatments at 12 h (**b**) and 24 h (**c**). (**d**) Heat map depicting clustering of the nine treatments by gene ontology (GO) terms associated with molecular and biological processes.

Significant differentially expressed genes (DEGs), defined here as p > 0.001 with a fold change > 2, were compared across the nine RNA-Seq treatments (Fig. 2). Three libraries were generated for each treatment and time points (note: only two libraries were generated for hatchling), but due to the high level of similarity between the replicates (Fig. S1) they were pooled for comparative purposes. A matrix comparing the total number of DEGs between treatments revealed that from the moment of hatching (0–30 min) through the first 24 h of development there is a significant increase of transcriptional activity over time (Fig. 2a). Of the different comparisons the largest difference between treatments was between the hatchling light organs and 24 h aposymbiotic animals exposed to the modeled microgravity conditions with 3091 DEGs. Although there was a dramatic increase in the overall number of transcripts between 12 and 24 h under both gravity and modeled microgravity conditions, the relative abundance of shared transcripts (APO, 17% at 12 h and 15% at 24 h; SYM, 12% at 12 h and 11% at 24 h) between the gravity and modeled microgravity did not extensively change (Fig. 2b). There were very few shared transcripts between all treatments with only 12 at 12 h and 44 at 24 h and were primarily unclassified with a few associated with ATP synthesis functions (Fig. 2b). The vast majority of the DEGs were unique to each treatment (Fig. 2b).

**Figure 3:**
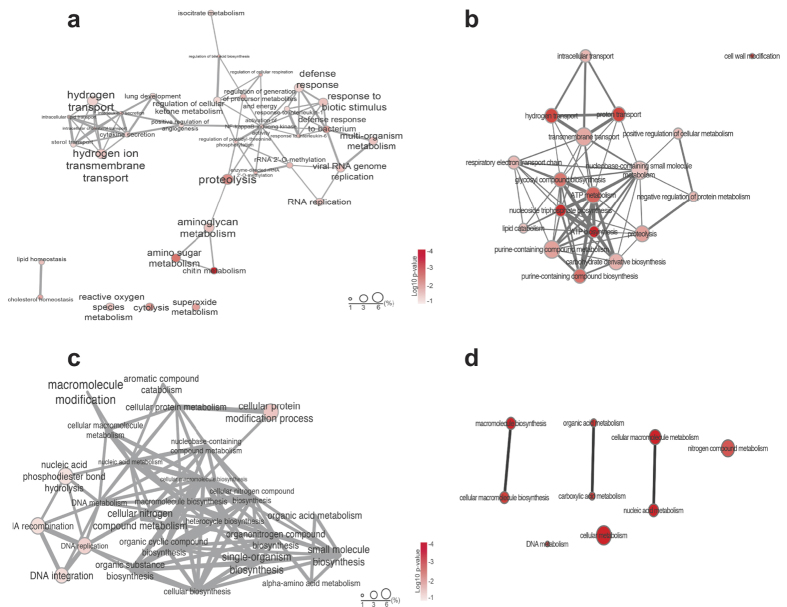
Gene Ontology (GO) network representing the differentially expressed GO categories under modeled microgravity or gravity controls at 24 h. Interactive networks of significantly enriched GO categories in aposymbiotic (**a**) or symbiotic (**b**) transcriptomes as well as depleted GO categories in aposymbiotic (**c**) and symbiotic (**d**) animals. Colors reflect log10 p-values. Circles represent proportion of the GO terms in the UniProt database. Higher frequencies (%) implies more general terms, whereas lower more specific ones. Highly similar GO terms are linked by edges in the graph, and the line width indicates the degree of similarity. Spatial placement of the nodes was computed by a ‘force-directed’ layout algorithm in Cytoscape that aims to keep the more similar nodes closer together.

A gene ontology (GO) enrichment and depletion analysis on the DEGs was conducted to assess which pathways were differentially represented within the nine treatments (Fig. 2d; Table S1). GO analysis revealed that the most significant DEGs were affiliated with the categories molecular function (MF) and biological processes (BP) including primarily enzymatic activities, such as hydrolase transferase, and catalytic activities (Fig. 2d). In all microgravity treatments there was an enrichment of cellular components (CC) GO terms associated with organelle synthesis, particularly with the mitochondria (Table S1). To visualize the frequency and interconnectivity of the enriched and depleted BP GO terms within the RNA-Seq treatments, interactive graphs were generated with REVIGO (Fig. 3; Fig. S2; Table S1). Results indicated that at 12 h in aposymbiotic animals there was an enrichment of GO terms primarily associated with purine nucleotide biosynthesis (Fig. S2a; Table S1), whereas 12 h symbiotic animals were enriched in GO terms associated with oxygen transport, oxidoreductase, antioxidant and reactive oxygen activity (Table S1; Fig. S2b). By 24 h the diversity of GO terms in both apo- and symbiotic animals was increased under microgravity conditions (Fig. 3). In aposymbiotic animals there were enriched transcripts with functions associated with hydrogen transport, cytokine secretion, chitin metabolism and reactive oxygen species (Fig. 3a), whereas depleted functions in cellular nitrogen metabolism and DNA metabolisms (e.g. recombination, replication; Fig. 3c). In symbiotic animals, however at 24 h, the enriched GO terms (Fig. 3b) included nucleotide processing and ion transport activities, whereas the depleted GO terms were associated with small molecule metabolism and ion/anion binding (Fig. 3d; Table S1).

### Changes in the innate immune and oxidative stress response in apo- and symbiotic animals under modeled microgravity conditions at 12 h

**Figure 4:**
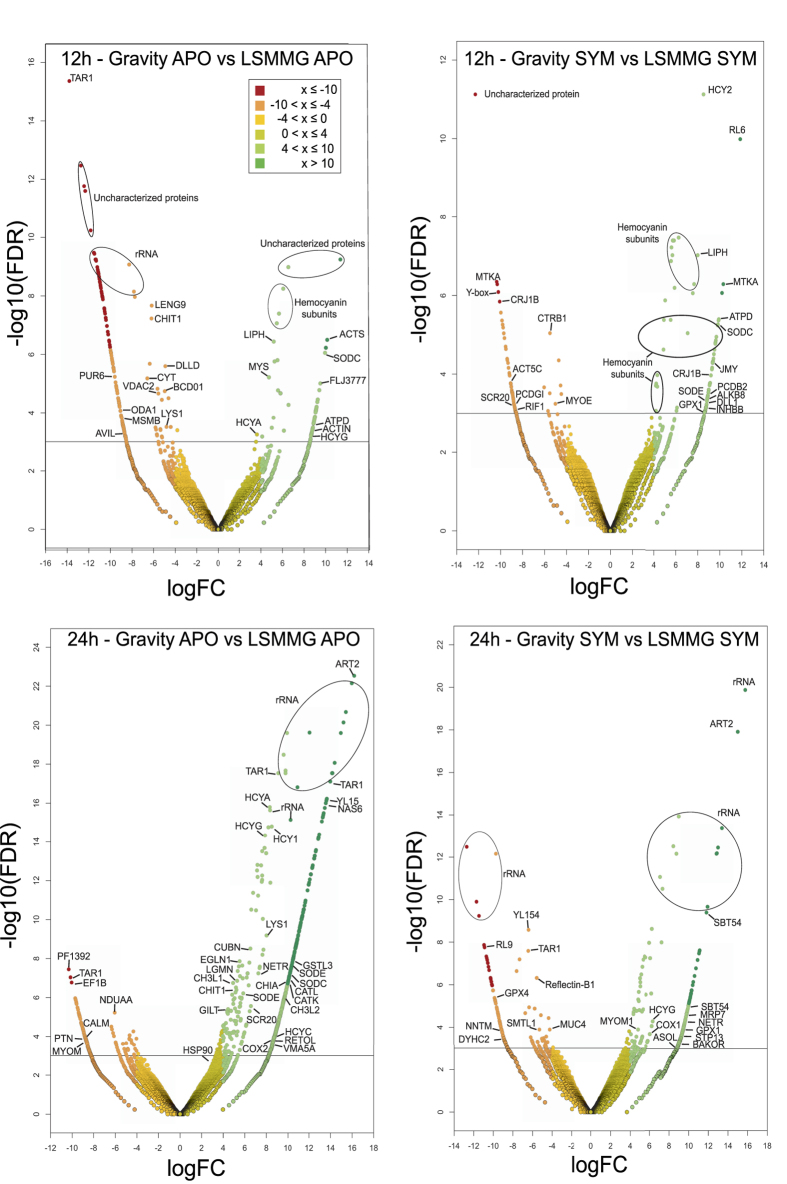
Volcano plots visualizing the global transcriptional changes between gravity and low shear modeled microgravity (LSMMG) conditions in apo- (APO) and symbiotic (SYM) light organs. All TMM-FPKM normalized transcripts were plotted and each circle represents one gene. Note genes labeled “hemocyanin” could not be classified to a specific subunit. The log fold change is represented on the x-axis whereas the –log10 of the false discovery rate (i.e., p-value) is on the y-axis. Grey lines represent a corrected (FDR) p-value = 0.001. Descriptions of the significant differentially expressed genes are listed in Table 2.

Animals exposed to modeled microgravity conditions for 12 h post hatching showed an increase in the number of genes associated with innate immune responses in both apo- and symbiotic animals, primarily transcripts associated with hemocyanin and lysosomal proteases. Although primarily known for its role in oxygen binding, molluscan hemocyanins exhibit phenol oxidase activities, which have been shown to have antimicrobial functions and play a role in the host innate immune response^48^. Two isoforms are present within *E. scolopes*^49^, which are comprised of seven to eight functional units^50^. Transcripts associated with both isoforms were observed under low shear modeled microgravity conditions in both apo- and symbiotic animals (Fig. 4; Fig. S3). There were, however, innate immune response transcripts expressed under the aposymbiotic gravity controls that were not observed in the modeled microgravity conditions, such as the expression of leucocyte receptor cluster member 9 (Leng9). Leng9 belongs to the CCCH zinc finger family and has been shown to be involved in regulating the innate immune response in mice, specifically during LPS-stimulated macrophage activation (Liang *et al*., 2008). Other transcripts upregulated in aposymbiotic animals during normal gravity controls that were absent in the modeled microgravity at 12 h include the proteases lysozyme (LYS1) and chitotriosidase (CHIT1). Chitotriosidase has been previously identified in *E. scolopes* during the first three hours of the symbiosis when *V. fischeri* are forming aggregations along the surfaces of the CEA^51^. The cells of the CEA express chitotriosidase, which metabolizes chitin to generating chitobiose a chemoattractant for *V. fischeri* to begin the colonization process^29^,^51^. No significant expression of the lysozyme or chitotriosidase was observed in symbiotic animals at 12 h, when colonization of the light organ has already been established, in either gravity or modeled microgravity conditions.

There was also an increase in the expression of oxidative stress genes transcripts at 12 h in both apo- and symbiotic animals exposed to modeled microgravity (Fig. 4; Table 2). For example, Cu/Zn superoxide dismutase (SOD) genes were enriched in both apo- and symbiotic animals under modeled microgravity conditions (e.g. SODC). Symbiotic animals also showed an increase in the expression of an extracellular SOD and glutathione peroxidase under modeled microgravity at 12 h. No significant DEGs associated with oxidative stress were observed in the gravity controls of either apo- or symbiotic light organs.

### Attenuation of the innate immune and oxidative stress response in symbiotic animals under modeled microgravity by 24 h

Apo- and symbiotic animals exposed to modeled microgravity conditions showed extensive differences in significant DEGs by 24 h. In aposymbiotic animals there was an increase in the number of expressed transcripts associated with the innate immune response in modeled microgravity compared to 12 h, such as hemocyanin (HCY), lysozyme (LYS), chitotriosidase (CHIT1/CH3L1), cathepsins (CATK/CATL), gamma-interferon-inducible lysosomal thiol reductase (GILT; Fig. 4c; Table 2). In the gravity controls, however, the DEGs associated with the innate immune response at 12 h were no longer expressed in aposymbiotic animals at 24 h. In modeled microgravity, the symbiotic animals showed no DEGs associated with the innate immune and oxidative stress response. Only hemocyanin G-type (HCYG) was upregulated in the symbiotic animals under microgravity conditions, whereas it was not significantly different under the gravity controls (Fig. 4d; Table 2).

The same trend was observed for the expression of genes associated with oxidative stress (Fig. 4c; Table 2). In aposymbiotic animals exposed to modeled microgravity there was a pronounced increase in transcripts encoding superoxide dismutase (SODC, SODE), Egl-9 hypoxia inducible factor (EGLN1), and reticuline oxidase (RETOL). Reticuline oxidase catalyzes the production of hydrogen peroxide from hexose sugars and has been shown to play an important role in pathogen resistance^52^. There was also a significant increase in transcripts associated with the chaperone protein HSP90, which is differentially regulated under a wide range of stress conditions including oxidative stress and has been shown to serve as a post-translational modulator of nitric oxide synthase in corals^53^. In gravity controls, however, no genes associated with oxidative stress were differentially expressed at 24 h in aposymbiotic animals. In symbiotic animals only glutathione peroxidase continued to be significantly differentially expressed under microgravity conditions.

**Figure 5:**
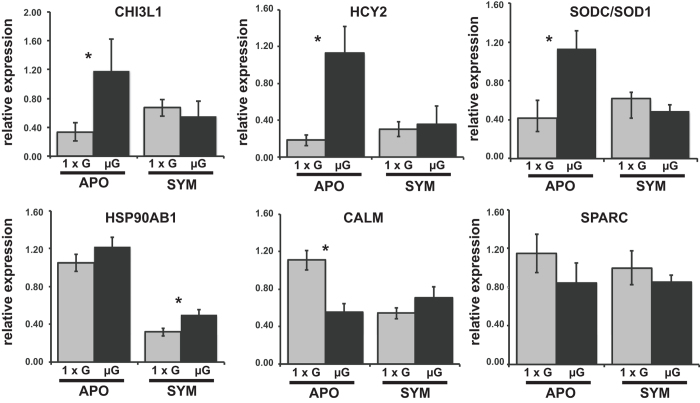
Quantitative analysis of mRNA derived from 24 h apo- (APO) and symbiotic (SYM) host light organs exposed gravity (grey; 1 × g) and low shear modeled microgravity (black; μG) conditions. The expression of selected genes from the RNA-Seq libraries was independently confirmed with quantitative real-time PCR. Differentially expressed genes included: chitinases (CHIT3L1), hemocyanin subunit 2 (HYC2), superoxide dismutase (SODC/SOD1), heat shock 90 beta (HSP90AB1), and calmodulin (CALM). The basement membrane-40 gene (SPARC) was not differentially expressed in the RNA-Seq libraries nor in the qPCR results. All data were normalized to the housekeeping gene actin.

The trends observed using RNA-Seq were independently validated with quantitative PCR (qPCR). Using animals that hatched from different egg clutches, selected DEGs from the RNA-Seq results were compared at 24 h under both gravity and modeled microgravity conditions (Fig. 5). Results correlated with the RNA-Seq results indicating that at 24 h the expression of genes associated with the host innate immune and oxidative response upregulated in aposymbiotic animals under modeled microgravity conditions were attenuated in symbiotic animals.

## Discussion

The mutualistic symbiosis between the bobtail squid *Euprymna scolopes* and its luminescent bacterium *Vibrio fischeri* provides an ideal opportunity to examine the impact of spaceflight on horizontally transferred host-microbe associations *in situ*. The results of this study show that the host squid can be inoculated and colonized with symbiotic *V. fischeri* in flight and that the symbiosis is highly amenable for space biology research. Further, RNA-Seq analysis of animals exposed to modeled microgravity, using HARV bioreactors, revealed pronounced differences in transcript expression of genes associated with the innate immune and oxidative stress response in the presence and absence of the symbiont *V. fischeri*. The results reveal the critical role that beneficial microbes may play in regulating the host transcriptome during modeled microgravity, specifically with regard to the innate immune and oxidative stress responses.

Although the flight experiments were limited to a single time point (23.5 h post-inoculation), the results demonstrate the malleability of the squid-vibrio system to monitor and examine the onset of colonization and bacteria-induced development during spaceflight. One advantage of the squid-vibrio system is that the symbiosis can be activated at any time point. The animals feed off of their internal yolk sac for the first 5–7 days after hatching and in the absence of symbiosis-competent *V. fischeri* the light organ does not undergo development^54^. At the start of the flight experiment the animals were 48 h-old but they not only showed successfully colonization, but also the activation of the host immune response. Interestingly, the presence of hemocytes-like cells in both flight and ground controls within the host crypts is earlier then the 48 h previously reported^41^ and potentially contradicts previous microgravity studies in which host macrophages have shown delays or decreased effectiveness in phagocytosis of pathogenic microbes^55^,^56^,^57^. Additional time points will be required to more fully examine the precise timeline associated with the infiltration of hemocytes into the crypt spaces under microgravity conditions as well as whether morphological changes associated with the crypts (e.g. cell swelling, microvilli density changes) are altered by space flight.

Analysis of the transcriptome of hatchling, apo- and symbiotic light organs under both microgravity and gravity conditions provided intriguing insight into genetic features of the host animal. With the lack of an available sequenced genome for *E. scolopes*, the 24 transcriptomes generated in this study were *de novo* assembled into a reference assembly that provided some important insight into the host organism. For example, 4.5% of the total transcripts presented more than one isoform with 487 transcripts exhibiting more than 10 different isoforms. The high level of observed isoforms is suggestive of extensive RNA editing within the host squid light organ. RNA editing is an important process that can change the sequence of message RNAs after transcription thereby potentially “recoding” the amino acid sequence and impacting the function of the protein^58^. A gene with isoforms that encode for the same protein may be subject to complex regulation to maintain a critical level of output and the various isoforms may be functionally specialized for different tissues or environmental conditions^59^. Cephalopods have been reported to exhibit extensive RNA editing with as much as 60% of the mRNA transcripts exhibiting recoding events, primarily within the nervous system^60^. These RNA editing events can be influenced by environmental factors, such as temperature, and may represent an important mechanism for animals to respond to environmental cues^58^,^61^,^62^. Here, the sequenced transcriptomes derived from the light organ suggest that, as in the nervous system, the light organ might also be a hot spot for RNA editing enabling the host to respond to *V. fischeri* colonization or changing environmental conditions. Further analysis of the assembled transcriptome coupled with ongoing genome sequencing efforts of *E. scolopes*^63^ will help elucidate the effects of environmental stresses, such as microgravity, on post-translational modifications within host animals.

Analysis of the transcriptome at the level of GO terms also revealed that in all microgravity treatments, regardless of the time point or presence of bacteria, there was an enrichment of intracellular organelle biological processes, in particular associated with mitochondria (Table S1). The absence of gravity results in enormous stresses on animal physiology at the cellular level^64^. A recent study examining organelle function in cardiomyocytes in simulated microgravity also showed an upregulation in genes and proteins associated with microchondrial processes as well as down-regulation of cytoplasmic translational mechanisms, including ribosomes and endoplasmic reticulum^65^. As mitochondria have been shown to be highly susceptible to stress-related damage and dysfunction in simulated microgravity^66^, the upregulation of light organ transcripts associated with these processes may be a mechanism for the host squid to maintain mitochondrial and other organelle homeostasis to help regulate cellular redox control during microgravity conditions, regardless of the symbiotic state of the host.

Although there were several microgravity-associated changes in the transcriptome that were uniform across treatments, there were some temporal and bacteria-specific changes as well. One of the most prominent changes in microgravity was the upregulation of oxygen transport protein, hemocyanin, in both the apo- and symbiotic animals at 12 h. The light organ is highly vascularized and oxygen is transported to the crypt spaces via the circulatory system with hemocyanin as the major respiratory protein^49^,^67^. The affinity for oxygen by hemocyanins is highly pH dependent and in symbiotic animals, which have high oxygen demands during the onset of luminescence^68^, there is a drop in pH due to the *V. fischeri* metabolism resulting in the release of oxygen in the crypt spaces. The upregulation of hemocyanin transcripts in microgravity conditions compared to gravity controls may reflect a response to potential hypoxic conditions, a potential problem during space flight for both animal and plant hosts^69^,^70^. In animals, hypoxia conditions can result in the inadequate oxygen transport causing numerous physiological conditions including dysregulation of innate immune system functions^71^.

Concomitantly, the increase in hemocyanin gene expression during microgravity may also reflect the anti-microbial properties of oxygen-transport proteins^48^. The hemocyanin of *E. scolopes* exhibits high levels phenol oxidase activities, including both catecholase and cresolase activities, which can be increased in the presences of bacterial serine-proteases^49^. The resulting quinone products of the phenol oxidase activity are anti-microbial and in conjunction with other host-derived products, such as nitric oxide and peptidoglycan recognition proteins, accumulate in the host-produced mucus that covers the CEA of the light organ^49^,^72^,^73^,^74^. Tolerance to these antimicrobials is critical for normal colonization by *V. fischeri*^75^ and *V. fischeri* has been shown to be resistant to these hemocyanin-derived products compared to other marine bacteria^49^. Together the antimicrobial cocktail present within the mucus may be playing an important role in selecting *V. fischeri* from the background microbial consortia present in the aggregate outside of the light organ.

By 24 h in microgravity conditions the upregulated expression of hemocyanin is attenuated and is only enriched in the aposymbiotic animals. This result may reflect, in part, the onset of the bacteria-induced regression of the CEA structures and subsequent decrease in mucus production of symbiotic animals by 24 h, suggesting that the upregulation of hemocyanin may have both antimicrobial and oxygen transport functions in microgravity conditions. Additionally, the decrease in hemocyanin production in the symbiotic animals at 24 h may also reflect changes in diel gene expression, as hemocyanin is differentially regulated through the day-night cycle of adult squid^76^. Additional time points will be needed to assess changes in the symbiont metabolism throughout the diel cycle in microgravity conditions and whether there are differential oxygen needs by *V. fischeri* under microgravity conditions.

In addition to hemocyanin, other genes typically associated with the innate immune response, primarily lysosomal proteins of the host were also differentially regulated in microgravity conditions. Comparative analysis of the 12 h and 24 h aposymbiotic transcriptomes revealed differential expression of protease transcripts, such as lysozymes, chitinases, lysosomal thiol reductases, and cathepsins (Fig. 4; Table 2). At 12 h in aposymbiotic animals maintained under normal gravity conditions there was an increase in expression in lysozyme and chitinases, which are known to help modulate the defense response of host organisms^51^,^77^,^78^. By 24 h, however, the expression of these proteases in gravity-treated aposymbiotic animals were no longer observed, but were significantly upregulated in microgravity conditions and were more broad including lysosomal thiol reductases, cathepsins and several different isoforms of chitinases (Fig. 4; Table 2). The upregulation of lysosomal proteases in microgravity has been seen in several other model systems, such as mouse and *C. elegans*, but has been primarily associated with muscle atrophy and autophagy^79^,^80^. In the symbiotic squid, however, there was no increase in expression of the lysosomal protease genes, suggesting that in the squid these proteases may be more essential to the immune response rather than muscle atrophy. Additionally, there is extensive evidence to suggest that lysosomal proteases can be regulated by both beneficial and pathogenic microorganisms^81^,^82^,^83^. The absence of differential expression of lysosomal proteases in the symbiotic animals may also reflect that by 12 h the colonization of the host light organ has already occurred. For example, during the initial 3 h of the symbiosis, *V. fischeri* changes the host gene expression to upregulate a host chitinases, which degrades chitin and leaves a chitobiose gradient for *V. fischeri* to chemotactically migrate through the pores on the surface of the light organ and colonize the crypt spaces^51^,^84^. These results suggest that at hatching the host animal is poised to receive the bacterial signal and that in the absence of *V. fischeri* the stress of being in a microgravity environment may trigger elements of the innate immune system as an adaptive response.

A similar effect was seen regarding the host oxidative stress response in the light organ. In plants and animals oxidative bursts are critical for the normal molecular exchanges between hosts and their associated microbes^85^. Additionally, microgravity has been shown to have a profound impact on the oxidative stress responses of eukaryotic hosts^86^,^87^,^88^. To help protect host tissues organisms secrete antioxidant enzymes, such as superoxide dismutases (SOD) and glutathione peroxidases (GPX)^89^. In the modeled microgravity conditions at 12 h, transcriptome analysis revealed an increase in Cu/Zn SOD transcripts in both apo- and symbiotic animals, whereas GPX transcripts were enriched only in the symbiotic animals. The antioxidant GPX appears to be constitutively expressed in the symbiotic light organs during modeled microgravity and likely serves as a housekeeping gene to help maintain oxidative environment in the host light organ. Additionally, the increase in SOD production could be correlated with the mitochondrial dysregulation observed in the host light organ in modeled microgravity conditions, as reactive oxygen species (ROS) are an abundant byproduct of oxidative phosphorylation^90^,^91^. However, by 24 h the expression of the SODs are significant only in the aposymbiotic animals, suggesting that compensating for mitochondrial dysregulation may not be the only function of the SODs.

In the *E. scolopes - V. fischeri* association reactive oxygen and nitrogen species (RONS) are critical for the initiation, selection and homeostasis of the symbiosis^28^,^91^. For example NO production, in conjunction with the production of hemocyanin, is critical for selection of *V. fischeri* in the aggregate outside of the light organ, whereas ROS are detected along the ciliated ducts that *V. fischeri* have to pass through to reach the crypt spaces^73^,^75^. Colonization of the light organ attenuates the RONS signals in the light organ^73^,^92^ and therefore *V. fischeri* may be critical for controlling the oxidative environment of the light organ during microgravity conditions.

The data provided here suggest that the presence of symbiotic bacteria help maintain homeostasis of the innate immune and oxidative environment of the host animal during modeled microgravity conditions. The differential expression of innate immune and stress genes in aposymbiotic animals at 12 and 24 h suggest that the light organ appears to be ready to respond to microbes and begin the selection process of culling *V. fischeri* from the environment. In the absence of that bacterial signal the stress of microgravity might serve as a physical cue for the aposymbiotic animals to alter gene expression and activate pathways that are typically associated with early colonization events of the symbiosis. Both chemical and physical stresses have been viewed as important contributors to the normal physiology and colonization of host tissues by beneficial microbes^85^. As microgravity represents a unique stress that terrestrial organisms have not previously encountered, the aposymbiotic host organisms might be activating these normal cellular response mechanisms typically associated with the onset of symbiosis, to potentially try to “adapt” to the novel environment. In the absence of the *V. fischeri* symbiont extended exposures to protease and oxidative stress responses may have negative health effects on the host animal.

In summary, the use of the *E. scolopes – V. fischeri* symbiosis provides an ideal platform to examine how healthy beneficial associations with microbes are initiated, regulated and potentially altered during space flight. Additional time points regarding how the host transcriptome changes in the presence and absence of the symbiont in microgravity conditions are essential to more fully elucidate the role beneficial microbes may play in maintaining homeostasis during space flight. Interestingly, at these two time points (12 and 24 h) the differentially expressed transcripts in the aposymbiotic animals were primarily associated with the onset and selection of *V. fischeri* from the environment. Transcriptome analysis revealed that although all elements of the innate immune response (e.g. NF-kB, Toll-like pathways) were observed in the host animals they were not differentially expressed during microgravity. A more comprehensive assessment, especially during the early colonization of the light organ (e.g. 3–6 h), may reveal whether other aspects of the immune system or other bacterial-modulated pathways are either positively or negatively impacted by microgravity. Lastly, this study provides an important first look into the molecular mechanisms underlying how beneficial microbes may help modulate the host response to microgravity, thereby helping to maintaining the health of the host tissues during space flight.

## Methods

### Ethics Statement

The University of Florida Institutional Animal Care and Use Committee (IACUC) was contacted prior to the start of the experiments but approval was not required, as invertebrates are not considered regulated animals. Prior to dissection animals were anesthetized in a 1:1 solution of 0.37 M MgCl_2_ and filtered seawater.

### General Procedures

A colony of adult *E. scolopes* was maintained in recirculating aquaria within an environmental growth chamber at 23 °C and a 12 h light/dark cycle. Clutches of eggs were removed from the adult tanks and incubated separately in individual aquaria for their full developmental cycle (≈21 days). After hatching, the juvenile squid were washed with filtered-sterilized seawater (FSW) and either maintained aposymbiotic (i.e., no symbiosis competent bacteria) or rendered symbiotic. For symbiotic treatments, animals were inoculated with 1 × 10^5^ cells of *V. fischeri* ES114 per ml of FSW. The concentration of *V. fischeri* was determined spectrophotometrically (A_600 nm_) as an OD of 1 corresponds to 3 × 10^8^ cells per ml of culture as previously determined by plate counts^93^. In some experiments the light organs were dissected from the host, homogenized in FSW and plated on SWT media to monitor the CFUs recovered from the different treatments as previously described^46^. In all treatments the onset of symbiosis was monitored using a photometer (GloMax 20/20 Luminometer, Promega, Corps., Madison, WI).

### Spaceflight experiments

The symbiotic partners, *E. scolopes* and *V. fischeri*, were launched aboard the space shuttle during missions STS-134 (May 16, 2011) and STS-135 (July 8, 2011) as part of the Commercial Reusable Experiments for Science & Technology (CREST-1) program. Note: the LMA hardware was processed for late loading on the space shuttle and the animals were 36 h-old at the time of launch. The partners were housed within in a liquid mixing apparatus (Fig. 1e; LMA; Instrumentation Technology Associates, Downington, PA), which contains three distinct compartments for the host squid, *V. fischeri*, and fixative (2.5% glutaraldehyde in a 0.2 M Sodium Phosphate buffer with 0.14 M Sodium Chloride pH 7.4). A total of six LMAs were flown during the two shuttle missions for a total of three symbiotic animals and three aposymbiotic controls. Approximately 12 h post launch, astronauts activated the LMAs by adding a concentrated *V. fischeri* solution to the squid compartment so that the final concentration was 1 × 10^5^ cells per ml of FSW. The aposymbiotic controls were inoculated with only FSW. After 23.5 h of incubation, the experiment was terminated and the host compartment was flooded with fixative (see below). The LMAs were maintained at room temperature for the duration of the experiment until the return of the shuttle to Earth (STS-134, June 1, 2011; STS-135, July 21, 2011). Replicate ground experiments were conducted for both space flight experiments.

### Transmission electron microscopy

To characterize the ultrastructural morphology of the crypt epithelial spaces of animals exposed to spaceflight conditions, animals were fixed in the LMA at a final concentration of 2.5% glutaraldehyde/2.5% paraformaldehyde solution containing 0.1 M sodium cacodylate with 0.45 M NaCl, pH 7.4 at room temperature. After landing, animals were processed as previously described^37^. Briefly, animals were rinsed in cacodylate/NaCl buffer, post-fixed for 45 min with a 1% osmium tetraoxide solution, and dehydrated with a standard ethanol gradient. Animals were then infiltrated with propylene oxide and accelerated Spurr resin and finally embedded in Spurr at 60 °C for 24 h. Thin sections were stained with uranyl acetate and lead citrate and visualized with a Hitachi H-7000 transmission electron microscope at the UF Interdisciplinary Center for Biotechnical Research.

### Modeled microgravity treatments

To simulate a low-shear modeled microgravity environment (LSMMG), rotary culture systems were used with 50-ml volume high aspect ratio vessels (HARVs; Synthecon, Houston, TX) at 13 rpm. To model the microgravity environment, HARVs were rotated around a horizontal axis, whereas gravity (1 × g) controls were rotated around a vertical axis. For the symbiotic treatments the HARVs were filled with FSW containing 1 × 10^5^ cells per ml of *V. fischeri* (unless otherwise indicated), whereas aposymbiotic treatments contained only FSW. The animals were added to the HARVs through an opening on the surface of the HARVs and then sealed. A semipermeable membrane provided aeration for the host and symbiont. Animals (between 4 and 5) were incubated in each HARV for either 12 or 24 h then immediately flash frozen in liquid nitrogen. For comparison purposes a subset of animals were collected immediately after hatching and frozen in liquid nitrogen.

### RNA isolation and transcriptome sequencing

For each treatment RNA was extracted from 10 dissected light organs using the RNeasy Kit (Qiagen, Valencia, CA). The manufacturer’s protocol was slightly modified as light organs underwent bead beating using a 6-mm stainless steel bead and then transferred to a QIAshredder column (Qiagen, Valencia, CA) before completing RNA extraction. The recovered RNA was DNAse-treated using a TURBO DNA-free™ Kit (Life technologies, Carlsbad, CA) and quantified using a Qubit 2.0 fluorometer (Life technologies, Carlsbad, CA) and normalized between treatments. RNA quality was assessed on an Agilent 2100 Bioanalyzer using a RNA 6000 Nano Kit (Agilent Technologies, Palo Alto, CA). High quality RNA underwent polyA selection and cDNA library synthesis using the NEBNext^®^ Ultra™ RNA Library Prep Kit for Illumina (New England Biolab, Ipswich, MA) and sequenced with the Illumina NextSeq500 platform, generating 2 × 150 bp paired end reads. All reads have been deposited in the NASA GeneLab database as well as at NCBI (bioproject SAMN06159576).

### Transcriptome assembly and evaluation

Initial FASTQ quality assessment, demultiplexing, and adapter trimming was performed using BaseSpace software (Illumina, San Diego, CA). A second quality filtering was performed with Trimmomatic^94^ using default parameters. Quality filtered reads were pooled together and *de novo* assembled using Trinity v. 2.0.6 95,96. Additionally, we performed three different assemblies in Trinity using *in silico* read normalization^97^ with *–normalize_max_read_cov* set to 50 and *–min_kmer_cov* set to 1 (I) or 2 (II), whereas for the third assembly (III) digital normalization was not used and set *–min_kmer_cov* to 1. *De novo* assemblies were evaluated with a reference-free transcriptome assembly evaluation measure using DETONATE v. 1.9^98^. The *in silico* normalized assembly with *–min_kmer_cov = 2* (II) reported the highest score, therefore was chosen as the targeted assembly for further analysis.

### Transcript quantification

Quality filtered reads from each individual library were subsequently mapped back to the candidate assembly using Bowtie for transcript quantification and gene expression analysis^99^. RSEM integrated in Trinity was used to generate read counts^100^. As RNA-Seq is a relative measurement, not an absolute one, a between sample normalization was necessary to compare transcripts abundance across samples^101^,^102^,^103^. FPKM normalization was performed on the number of quality-filtered reads that only match back to the reference transcriptome, which being *de novo* was assembled using only the same reads. Normalization of reads counts was performed by EdgeR v. 3.2^104^ as “Fragments Per Kilobase of transcript per Million mapped reads” (FPKM)^59^, normalized by a trimmed mean of M-values normalization method (TMM)^99^,^105^. Differential expression analysis was conducted using EdgeR and only differentially expressed transcripts with a *p* < 0.001 with a minimum fold change of two were included.

### Prediction of protein and annotation

Protein-coding sequences were predicted from the assembled transcriptome using TransDecoder v. 2014^96^ with default parameters, retaining ORFs that were at least 100 amino acids long. Assembled transcripts were screened for sequence homologies and corresponding Gene Ontology (GO) using BLASTX^106^ against the SwissProt (v.2015) and UniRef90^107^ databases. Gene ontology enrichment was conducted using a combination of Trinotate v. 2.0.6 (http://trinotate.github.io/) and the ‘GOseq’ R package in Bioconductor^108^ TransDecoder-predicted coding regions were also searched for sequence homologies using BLASTP^106^, within the same databases. Protein domains were identified via HMMER v. 3.1^109^ against the PFAM protein families database^110^. Additionally, signal peptides and transmembrane-spanning regions were predicted using SignalP v. 4.1^111^ and TMHMM v. 2.0^112^, respectively. rRNA structural genes were predicted using RNAmmer v. 1.2^113^. Finally, transcript sequences with relative overall annotations and gene/transcripts relationships with all 23 libraries were loaded into a SQLite database for exploring purposes using Trinotate.

### Gene ontology network generation and visualization

GO terms were filtered to remove redundancy and the top scoring elements were visualized in a network-based relationship using the web-based tool REVIGO (http://revigo.irb.hr/)^114^. Only significantly (p ≤ 0.001) enriched or depleted genes, as computed by GOSeq R package, were used as input in REVIGO with the following parameters: I) UniProt database; II) similarity = 0.7 database; III) SimRel as semantic similarity measure. The resulting networks were then imported into Cytoscape^115^ and visualized using a force-directed layout algorithm to convey the relationship between the nodes.

### Quantitative PCR

Reactions were prepared using the iTaq Universal SYBR Green One-Step Kit (Biorad, Hercules, CA) with 10 ng of total RNA for each reaction. Amplification and quanitification was completed using a SCX9600 Real Time System (Biorad, Hercules, CA). Primers were designed to targeted transcripts using Primer3^116^ and are listed in Table S2. β-Actin and 18 S rRNA gene served as references for comparison of expression between treatments. The reaction conditions included an initial incubation at 50 °C for 10 min then 1 min at 95 °C followed by 39 cycles of 95 °C for 10 s and 60 °C for 15 s. A melting curve of 95 °C for 10 s and 65 °C for 5 s was conducted. Reactions were performed in triplicate and three technical replicates were repeated for each treatment and primer set.

## Additional Information

**How to cite this article**: Casaburi, G. *et al*. Transcriptomic changes in an animal-bacterial symbiosis under modeled microgravity conditions. *Sci. Rep.* **7**, 46318; doi: 10.1038/srep46318 (2017).

**Publisher's note:** Springer Nature remains neutral with regard to jurisdictional claims in published maps and institutional affiliations.

## Supplementary Material

Supplementary Figures and Tables

Supplementary Dataset

## Figures and Tables

**Table 1:** Overview of recovered light organ transcriptome sequencing results from *E. scolopes* exposed to modeled microgravity conditions.

Treatment	Time Point	High-quality Reads	Mapped Reads^a^ (% mapped)	FPKM^b^
Hatchling	0 h	54.70 million	27.68 million (50%)	14.24
Gravity – APO^c^	12 h	48.22	23.95 (49.7)	16.78
Gravity – SYM^d^	12 h	56.74	30.52 (53.7)	15.34
LSMMG^e^-APO	12 h	34.38	16.94 (49.3)	18.08
LSMMG-SYM	12 h	49.92	27.74 (55.6)	15.94
Gravity - APO	24 h	35.80	21.04 (58.8)	17.17
Gravity - SYM	24 h	34.55	19.44 (56.3)	23.50
LSMMG-APO	24 h	48.57	25.90 (53.3)	21.43
LSMMG-SYM	24 h	66.23	34.14 (51.5)	15.37

^a^Reads mapped to individual contigs.

^b^Fragments per kilobase of transcript per million mapped reads reflecting the normalized average read depth.

^c^Aposymbiotic animals without exposure to bacteria.

^d^Symbiotic animals exposed to *V. fischeri*.

^e^Animals exposed to low-shear modeled microgravity.

**Table 2:** Selected genes differentially expressed in aposymbiotic (APO) and symbiotic (SYM) host squid during low shear modeled microgravity (LSSMG) conditions.

UniProt #	Gene/Protein	LSMMG		P-value APO/SYM
		APO	SYM	
DLLD_DANRE	*dld*/Delta-like protein D	−6.5	+8.6	6.61E-06/0.0009
PCDGI_HUMAN	*PCDHGB6*/Protocadherin gamma-B6	nd	−8.9	na/0.0002
PCDB2_PANTR	*PCDHB2*/Protocadherin beta-2	nd	+8.8	na/0.0009
CTRB1_LITVA	/Chymotrypsin BI	nd	−5.5	na/9.10E-06
SCR20_NOTSL	/S-crystallin SL20-1	nd	−8.9	na/0.0002
HCYG_SEPOF	/Hemocyanin, units G and H	+8.7	+9.2	0.0005/5.20E-05
GPX1_SCHMA	*gpx1*/Glutathione peroxidase	nd	+8.6	na/ 0.0009
SODC_CANAX	*sod1*/Superoxide dismutase [Cu-Zn]	+9.9	+9.8	8.80E-07 /5.52E-06
SODE_MOUSE	*sod3*/Extracellular superoxide dismutase	nd	+8.6	na/ 0.0008
**24 h exposure**				
HSP90AB1	*hsp90*/Heat shock cognate protein HSP90-beta	+2.1	nd	0.02/na
CALM_OREMO	*CAM*/Calmodulin	−8.4	nd	0.0005/na
MYOM1_APLCA	*myomod1/* Myomodulin neuropeptides 1	+3.9	−8.5	0.0006/0.0003
PTN_BOVIN	*PTN*/Pleitrophin	−8.7	nd	0.0001/na
NETR_MACMU	*PRSS12*/Neurotrypsin (Serine protease)	+9.8	+9.1	6.06E-07/0.0003
LYS_MERLU	/Lysozyme (1,4-beta-N-acetylmuramidase)	+9.8	nd	6.10E-07/nd
CHIA_RAT	*CHIA*/Acidic mammalian chitinase	+9.9	nd	2.47E-07/na
CHI3L1	*CHI3L1*/Chitinase-3-like protein	+5.3	nd	4.38E-08/na
CHI3L2	*CHI3L2*/Chitinase-3-like protein	+9.6	nd	1.33E-06/na
GILT_BOVIN	*IFI30*/Gamma-interferon-inducible lysosomal thiol reductase	+4.4	nd	3.92E-06/na
MRP7_HUMAN	*ABCC10*/Multidrug resistance-associated protein	+9.1	nd	0.0003/na
SPRC_CHICK	*ost-1/* Ostonectin - SPARC (secreted protein acidic and rich in cysteine)	nd	+7.6	na/0.001
VMA5A_HUMAN	*VMA5A*/von Willebrand factor A domain-containing protein	+8.3	nd	0.0005/na
CATK_BOVIN	*CTSK*/Cathepsin K	+4.9	nd	1.77E-07/na
CUBN_HUMAN	*CUBN IFCR*/Cubulin	+9.5	nd	2.73E-06/na
SCR20_NOTSL	/S-crystallin SL20-1 lens polypeptide	+8.8	nd	8.44E-05/na
DYHC2_TRIGR	*DYH1B*/Cytoplasmic dynein 2 heavy chain 1	−8.9	nd	0.0003/na
HYDIN_MOUSE1	*hydin*/Hydrocephalus-inducing protein (isoform 1)	−8.6	nd	0.0007/na
HYDIN_MOUSE2	*hydin*/Hydrocephalus-inducing protein (isoform 2)	+4.6	nd	0.0008/na
GPX1_SCHMA	*gpx1*/Glutathione peroxidase	na	+8.9	na/0.0007
EGLN1_RAT	*egln1*/Egl nine homolog - Hypoxia-inducible factor prolyl hydroxylase 2	+8.7	nd	0.0001/na
RETOL_ARATH	/Reticuline oxidase-like protein	+8.4	na	0.0004/na
SODC_CANAX	*sod1*/Superoxide dismutase [Cu-Zn]	+10.3	na	2.54E-08/na
SODE_CAEEL	*sod3/* Extracellular superoxide dismutase [Cu-Zn]	+4.9	na	5.70E-05/na
HCYG_ENTDO	*ODHCY*/Hemocyanin G-type – Iso1	+7.9	+6.3	4.67E-15/5.36E-05
