# Supplementary material for: Transcriptomic changes in an animal-bacterial symbiosis under modeled microgravity conditions
Source: Sci Rep. 2017 Apr 10;7:46318. doi: 10.1038/srep46318 (PMC5385879; doi:10.1038/srep46318)
Supplement: Supplementary Figures and Tables [file srep46318-s1.pdf]

Supplemental Material

Transcriptomic changes in an animal-bacterial symbiosis under modeled microgravity conditions

Giorgio Casaburi, Irina Goncharenko-Foster, Alexandrea A. Duscher and Jamie S. Foster*

Department of Microbiology and Cell Science, University of Florida, Space Life Science Lab, Merritt Island, FL, USA.

*Corresponding Author

E-mail: jfoster@ufl.edu; Tel: 321-525-1047

Figure S1

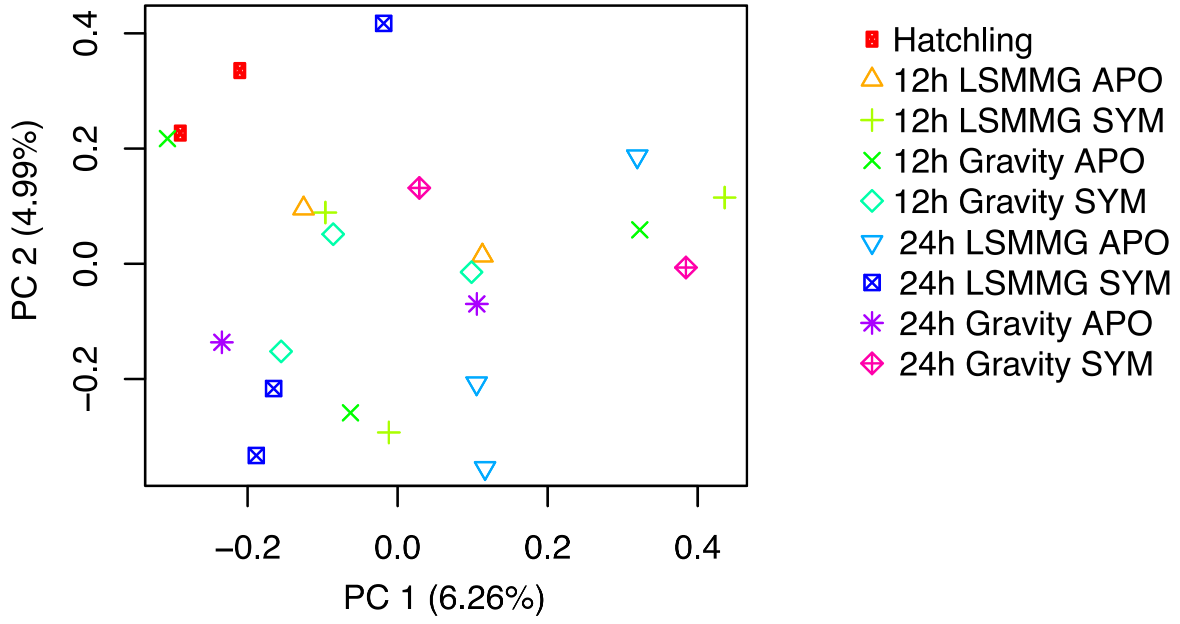

Figure S1. Principal component analysis of all RNA-Seq libraries generated in this study demonstrated low overall variability between treatments (PC1 5.17%). Treatments included aposymbiotic (APO) and symbiotic animals incubated for 12 and 24 h under modeled microgravity and gravity conditions.

Figure S2

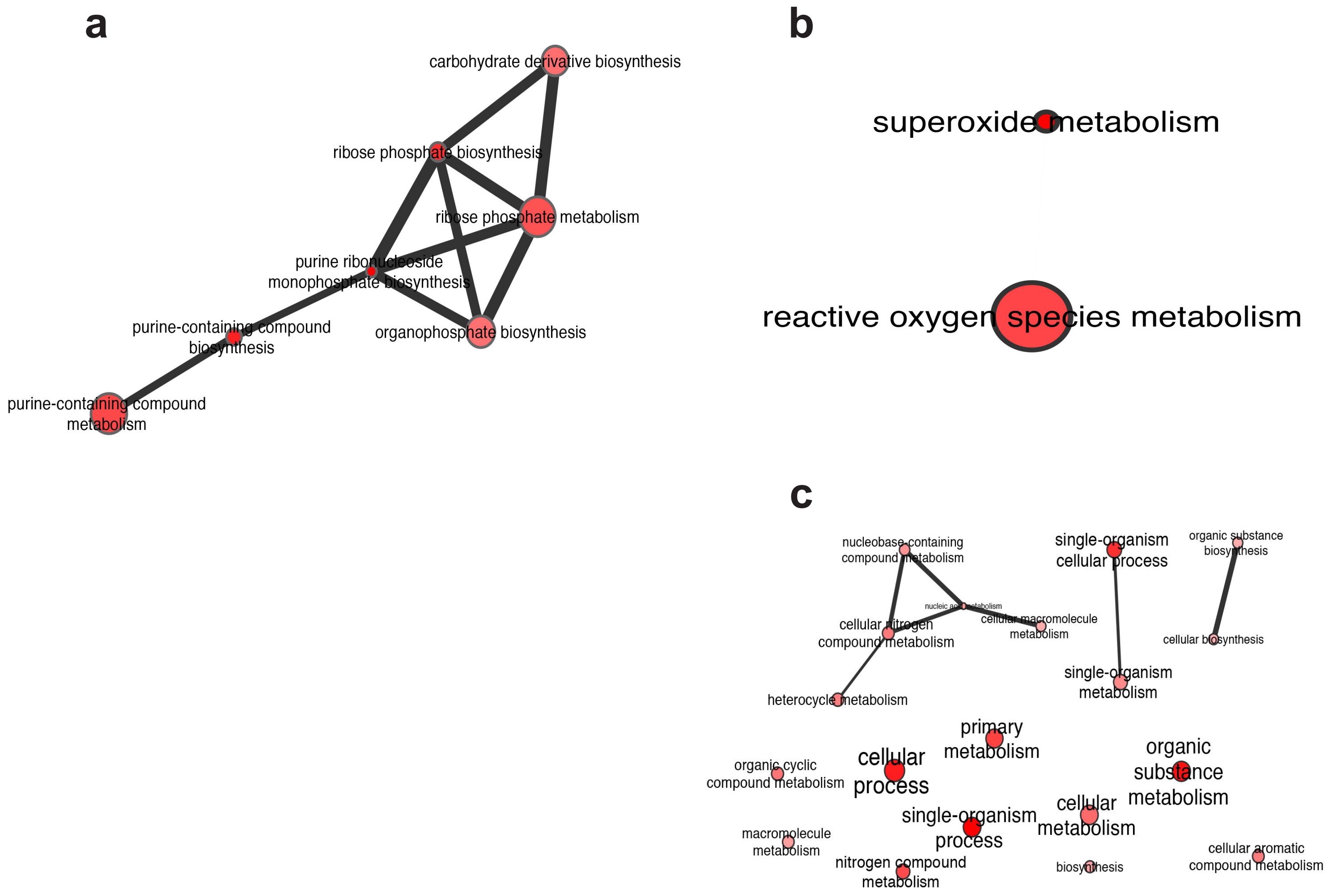

Figure. S2. Gene Ontology (GO) network representing the differentially expressed GO categories under modeled microgravity or gravity controls at 12 h. Interactive networks of significantly enriched GO categories in aposymbiotic (a) or symbiotic (b) transcriptomes as well as depleted GO categories in symbiotic (c) animals (note: no GO terms were depleted in aposymbiotic animals at 12 h). Colors reflect log10 p-values. Circles represent proportion of the GO terms in the UniProt database. Higher frequencies (%) implies more general terms, whereas lower more specific ones. Highly similar GO terms are linked by edges in the graph, and the line width indicates the degree of similarity. Spatial placement of the nodes was computed by a ‘ force-directed’ layout algorithm in Cytoscape that aims to keep the more similar nodes closer together.

Figure S3

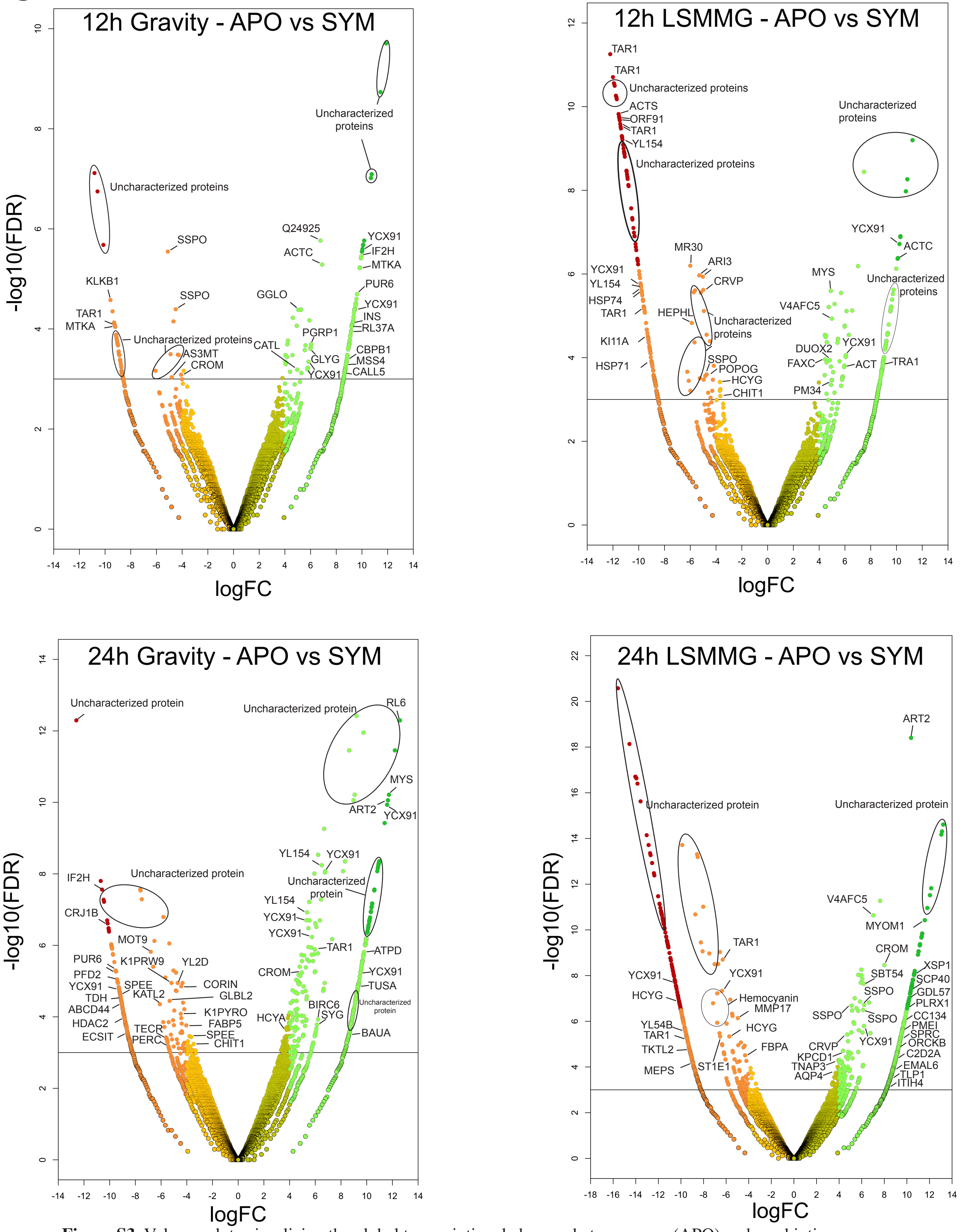

Figure S3. Volcano plots visualizing the global transcriptional changes between apo- (APO) and symbiotic (SYM) light organs under gravity and low shear modeled microgravity (LSMMG) conditions at 12 and 24 h. All TMM-FPKM normalized transcripts were plotted and each circle represents one gene. Note genes labeled "hemocyanin" could not be classified to a specific subunit. The log fold change is represented on the x-axis whereas the –log10 of the false discovery rate FDR (i.e., p-value) is on the y-axis. Grey lines represent a corrected (FDR) p-value = 0.001. Descriptions of the significant differentially expressed genes are listed in Table 2.

Target Gene	Direction	Sequence (5' → 3')	Amplicon Size	T_m (°C)	Reference
β-Actin	Forward	ATGTTCCCCGGTATTGCTGA	115 bp	56.4	Pankey et al., 2014
	Reverse	CGCCGATCCAGACAGAGTAT		56.3	
18S rRNA gene	Forward	CGTTTTCCTCGATCAAGAGC	77 bp	53.7	Pankey et al., 2014
	Reverse	CATCGTTTACGGTCGGAACT		54.8	
CHI3L1	Forward	GGAGTGATGACGTGGTCGTT	103 bp	60.0	This study
	Reverse	AATCCCCTGAAAGTGTGCGA		59.6	
HCY2	Forward	AAATGTCGACGGAAGCCCAC	82 bp	61.0	This study
	Reverse	AACAGCGTGATGTTTAGCGG		59.2	
HSP90AB1	Forward	GGCAAAACTTTGGTCTGCGT	183 bp	59.9	This study
	Reverse	ACAGCAGGGAGATTCCACAA		58.9	
SODC	Forward	TGTGATGATACAGCAGGCCA	71 bp	59.1	This study
	Reverse	GCAAGGGAGAATCCCCAGAC		60.1	

Table S2. Primers used for quantitative PCR to confirm selected RNA-Seq results.
